# Women’s perspectives on motivational factors for lifestyle changes after gestational diabetes and implications for diabetes prevention interventions

**DOI:** 10.1002/edm2.248

**Published:** 2021-03-19

**Authors:** Lisbeth Ørtenblad, Diana Høtoft, Rubab H. Krogh, Vibeke Lynggaard, Jens Juel Christiansen, Claus Vinther Nielsen, Anne‐Mette Hedeager Momsen

**Affiliations:** ^1^ DEFACTUM – Public Health and Rehabilitation Research Central Denmark Region Aarhus Denmark; ^2^ Department of Public Health Aarhus University Aarhus Denmark; ^3^ Department of Gynaecology and Obstetrics Gødstrup Hospital Herning Denmark; ^4^ Steno Diabetes Center Aarhus Health Promotion Research Aarhus Denmark; ^5^ Cardiovascular Research Unit Department of Cardiology Gødstrup Hospital Aarhus Denmark; ^6^ Department of Medicine Gødstrup Hospital Aarhus Denmark; ^7^ Section for Clinical Social Medicine and Rehabilitation Gødstrup Hospital Aarhus Denmark

**Keywords:** gestational diabetes, health care delivery, prevention of diabetes

## Abstract

**Introduction:**

Gestational diabetes mellitus (GDM) is a common complication in pregnancy and constitutes a public health problem due to the risk of developing diabetes and other diseases. Most women face barriers in complying with preventive programs. This study aimed to explore motivational factors for lifestyle changes among women with a history of GDM and their suggestions for preventive programs.

**Methods:**

This study used a qualitative approach in six focus group interviews with a total of 32 women. The selection criteria were time since onset of GDM, including women diagnosed with GDM, six months and five years after GDM, diagnosed and not diagnosed with diabetes. Inductive analysis was performed.

**Results:**

The women reacted with anxiety about their GDM diagnosis and experienced persistent concerns about the consequences of GDM. They were highly motivated to take preventive initiatives, but faced major adherence challenges. The demotivating factors were lack of time and resources, too little family involvement, lack of knowledge and social norms that may obstruct healthy eating. A powerful motivational factor for complying with preventive strategies was the well‐being of their children and partners.

**Conclusions:**

Preventive initiatives should be rooted in the women's perception of GDM/diabetes and based on their experiences with barriers and motivational factors. The well‐being and the quality of life within the family are dominant motivational factors which offer powerful potentials for supporting the women's coping capability. Further, there is a need to be responsiveness to the women and their families even a long time after the onset of GDM.

## Abstract 2

Even a long time after GDM the women are worried about the health consequences of the diagnosis and all though motivated to take preventive actions they experience barriers to adhere to the recommended lifestyle changes. To better overcome such barriers the women's recommend preventive initiatives to be rooted at the core of their everyday life and to include their families. It is important to pay attention to the women for a long time after the GDM diagnosis and to base preventive programs within the women's perspectives of GDM/diabetes and preventive initiatives.

**Novelty statement**

**What is already known?**

- Women experience barriers to obtaining healthier lifestyles after GDM, especially lack of time, energy and social support.

**What this study has found?**

- The women experienced long‐term (min. five years) worries of the health consequences of the GDM diagnosis and were motivated to participate in preventive programs over a similar long period of time.
- The women's suggestions concerning preventive initiatives were rooted at the core of their everyday life which strengthened their sense of coherence.

**What are the clinical implications of the study?**

- It is important to pay attention to the women for a long time after the GDM diagnosis and to base preventive programs within the women's perspectives of GDM/diabetes and preventive initiatives.

## 1 INTRODUCTION

Gestational diabetes mellitus (GDM) is common during pregnancy with a prevalence in Europe between 2–6%^1^, ^2^, ^3^ and <24% worldwide, depending on the population and diagnostic criteria.^4^ An increase in GDM has been observed worldwide for decades, due to the increase in obesity and older age at conception.^5^, ^6^, ^7^ GDM is a strong predictor for developing type 2 diabetes for the mother and child^8^, ^9^: 50% will develop diabetes within 10 years, and the women´s lifetime risk is seven times higher than among women without a history of GDM.^2^, ^9^, ^10^, ^11^ The offspring has eight times increased risk of developing diabetes and doubled risk of overweight later in life.^2^ Moreover, partners to women with GDM have increased risk of developing diabetes,^12^ ^,^ ^13^ suggesting that diabetes also is socially embedded in the sense that not only biological but also patterns of behaviour related to, for example, lifestyle factors in the families and social relations play an important role.

Prevention of diabetes is possible using lifestyle changes.^14^, ^15^, ^16^ Systematic reviews found promising strategies including promotion of physical activity, healthy diet and weight control. However, barriers to lifestyle changes after GDM have been identified, especially lack of time, energy and social support, which may lead to poor adherence.^15^, ^17^, ^18^, ^19^ Postpartum care following GDM is characterized by uncertain division of responsibilities in the healthcare sector, and women experience little attention regarding their needs, which results in dropping out from programs following GDM.^20^

Women's experiences with GDM and barriers to comply with preventive interventions have been studied, but there is still a need to explore what motivates the women to attend preventive programs and maintain lifestyle changes, especially over time. This study aimed to explore the perspectives and motivational factors for initiating and maintaining lifestyle changes among women with a history of GDM. The purpose was to initiate a future preventive program targeting women's needs in a local Danish setting. In the context of the study, we defined motivation as a concept referring to a process which engender behaviour from intentions to actual actions with the aim of reaching certain goals; and motivational factors were defined as conditions which provide meaning to do an effort to reach certain goals.

## 2 MATERIALS AND METHODS

### 2.1 Design, participants and data collection

The study applies qualitative method, using focus group interviews as data collection method. The method allows the participants’ to exchange experiences and views, thus contributing to knowledge about the extent of consensus and diversities among the participants.^21^ Accordingly, the focus group interviews provided more than the sum of individual interviews because the participants questioned and spoke to each other.

Six focus group interviews were conducted. To explore time‐related changes, time from GDM diagnosis to participation in the interview was important, and the study covered a 5‐year period after the onset of GDM. One group included pregnant women diagnosed with GDM; one group included women six months after GDM; two groups included women five years after GDM, all diagnosed with diabetes; two groups included women five years after GDM without diabetes. The participants were recruited from the Department of Obstetrics at the local hospital where the study took place. Women characterised by the inclusion criteria defining the respective focus groups were selected consecutive from the department register until 10–12 persons for each group was achieved. They were contacted by telephone for invitation to participate in the focus group interviews and upon acceptance, a written invitation explaining the purpose of the study and the use of data was mailed to them. 32 of the listed 67 potential participants, agreed participation. The main reasons for refusal were unanswered calls or lack of time. A few women mentioned lack of resources or insufficient knowledge of Danish. Most participants were above 30 years and had two children. About half of the women had experience with GDM in more than one pregnancy Table 1.

**TABLE 1:** Participants’ characteristics

Characteristics	Category	Number of women
Number of children	0	5
	1	6
	2	12
	3	9
Number of previous pregnancies with gestational diabetes mellitus	0	15
	1	10
	2	6
	3	1
Age	20–30 years	3
	31–40 years	14
	41–50 years	15

The focus group interviews took place at the local hospital. 4–7 women participated in each focus group. Rapport between interviewers and participants was established initiating the focus group interviews by informal conversations and the researchers introducing themselves and their reasons for doing the research project. The interviews were directed by a thematic, semi‐structured interview guide, focusing on experiences of being diagnosed with GDM; attitude to risk factors; attitude and experiences regarding barriers to and motivation for prevention of consequences of GDM; perceptions of meaningful and relevant preventive initiatives. Small tasks were used to stimulate discussions among the participants, for example cards with statements about prevention and lifestyle. To allow comparison across the groups, this approach was implemented in each group. Two of the female authors, who were trained and experienced within qualitative research, (LØ and DH) implemented the focus group interviews which were conducted in Danish. Each interview lasted approximately two hours, was recorded digitally and transcribed verbatim. Notes were written after the interview, focusing on the proceeding of the interview and interaction of the participants.

Table 2 presents the study setting.

**TABLE 2:** Description of the study setting

This study was performed at the Regional Hospital West Jutland, one of five hospitals in the central Denmark region. This hospital serves approximately 300,000 citizens who live in a rural area with six provincial municipalities. Approximately 150 women with GDM are treated annually at the hospital's Department of Obstetrics. The Danish healthcare system is funded by taxes and is provided free of charge with equal access to healthcare services for all people. According to the national guidelines, women with GDM receive specialized healthcare services with a focus on blood glucose levels, ultrasound imaging for the foetus and advice on lifestyle changes from doctors at outpatient clinics in local hospitals with obstetric departments. The postpartum follow‐up is provided by a general practitioner who is responsible for individual diabetes screening and guidance of lifestyle factors regarding prevention of diabetes. Preventive follow‐up programs are missing from the routine clinical set‐up as well as standardized referrals to these possible local programs.

### 2.2 Data analysis

The data analysis followed an inductive process based on a thematic analysis^22^ including four steps: (a) Becoming conversant with the data: The interview transcripts were thoroughly read by two authors (LØ, AMM), several times by one author (LØ) and briefly read by other authors (VL, RK, JJC). (b) Generating codes: Initial codes were generated from the data, tested by coding two interviews and adjusted by two authors (LØ, AMM), for example reduced from eight to six codes due to overlaps and clarification of definitions. The final codes were discussed and agreed upon by all authors (see Table 3 for a description of the codes) and coded by one of the authors (LØ). (c) Condensation: Data were coded and condensed. (d) Analysis: Generating themes, critical interpretation and synthesis.

**TABLE 3:** Code structure and contents

Code	Definition
Experience of being diagnosed with GDM	The women's thoughts, reflections, and experiences of having/having had the diagnosis. How do they feel about it? How does it affect them? How do they handle it? Knowledge about possible consequences?
Diabetes	The women's perspective on diabetes (their own, their social surroundings). The women's assessment of and knowledge of risk factors.
Prevention of diabetes and other consequences of GDM: Motivational factors Obstacles/challenges Adhering to new habits	The women's motives and incentives to prevention of consequences of GDM. The women's perception of challenges and barriers regarding prevention. What does it takes to maintain new habits?
Information and knowledge	From whom, where and when do the women receive information about GDM and prevention of its consequences? How is the information perceived regarding its practicability, relevance and utility? How should information be communicated to make sense and to be applicable?
Collaboration with healthcare professionals	Where do the women receive help/assistance and when during the course? Communication (how is it communicated, by who). The women's assessment of the collaboration, that is the women's need for assistance and collaboration (eg pre‐ and post‐GDM/childbirth).
Intervention suggestions: Substances Form Time, duration Auspices Accessibility/location Others	The women's requests, ideas and needs for an appropriate design of postpartum preventive interventions. What would best accommodate their needs?

Quotations are used throughout to illustrate the findings of the study. They were selected from a broad range of the participants to cover views among all participants. When perspectives from a specific focus group are critical to understand the importance of the finding the group—for example women five years after GDM with/without diabetes—is mentioned in the text.

NVivo 11.0 QSR software was used to handle the data. Consolidated criteria for reporting qualitative research (COREQ checklist) was used to provide a quality assessment of the reporting of the methodology.^23^

### 2.3 Ethics

The study was approved by the Danish Data Protection Agency (case number 1–16–02–180–17). Danish legislation requires no official ethical approval for studies not involving human or biological material (National Committee on Health Research Ethics). All potential participants were contacted by one of the researchers (LØ), who presented herselves and the reason for doing the research at this initial contact. Further, the study purpose and management of data were explained to all participants orally and in writing and informed consent were provided. The participants were anonymized. They received a modest gift voucher in appreciation for their participation.

## 3 RESULTS

The codes were condensed into four themes using the definition of motivation as previously described: experiences of GDM as motivating preventive strategies; experiences of demotivating barriers to prevention strategies; experiences of motivational factors for prevention strategies; suggestions for motivating preventive programs.

### 3.1 Experiences of GDM as motivating preventive strategies

Being diagnosed with GDM was an upsetting experience for most of the women, as told by one of them:

I was shocked. I had read about being overweight and physically inactive and I may fall within that group, but anyway, I was very upset and thought, oh my God, what did I do wrong? I was just pregnant, after all.

The women experienced a change of identity from being a healthy person with a normal pregnancy to being ill having to adjust their expectations over the course of pregnancy. Besides time‐consuming hospital appointments to manage their GDM, they had to adjust their daily routines to follow advice regarding diet and exercise. Consequently, GDM markedly changed their everyday life and self‐image.

The women were concerned about the consequences of GDM, especially for their unborn baby. Most women including those 5 years after GDM were aware of their own risk, whereas only a few were aware of the long‐term consequences for their child and partner.

Also, the women experienced the diagnosis as a psychological burden, for example:

I felt myself to be a failure. Honestly, are you not even able to be pregnant? It’s like it’s your own fault. You feel guilty about being too fat and not exercising.

The women blamed themselves for GDM due to their body weight and difficulties in complying with advice on lifestyle changes. Some felt ashamed and concealed the diagnosis. Further, the women experienced stigmatization in the healthcare system:

You are labelled as an overweight. I have met many doctors who only see it from this point of view. You are kind of stupid and wrong if you cannot eat as you are supposed to and that kind of stuff.

It appeared they felt reassured by being monitored at the hospital during their pregnancy, but as the quote illustrates they also experienced a moralistic attitude from the health service providers.

Although the women after childbirth were happy about being well again, they still experienced diabetes as a potential risk. This appeared in groups of women respectively six months and five years after their GDM diagnosis:

You kind of forget it [GDM/diabetes] after the childbirth. But it is still important. In the back of my mind I think, ‘I could be one of them’.

I was upset then [being diagnosed with GDM], and even now [five years after GDM] I keep on thinking, do I now have to struggle with that the rest of my life?

Thus, even many years after their GDM diagnosis the women continued to be concerned about the risk and were therefore motivated to prevent the long‐term consequences of GDM.

### 3.2 Experiences of demotivation barriers to prevention strategies

When talking about preventive strategies, all of the women mentioned the importance of diet and physical activity. They intended to live a healthy lifestyle after childbirth, but found it difficult to comply with the guidelines from healthcare professionals. They experienced a range of demotivating factors, analytically condensed into four sub‐themes: lack of time and resources; lack of family involvement; barriers to healthy eating; and lack of knowledge.

#### 3.2.1 Lack of time and resources

All the women stressed that they, as new mothers, had their focus elsewhere; several were mothers with two to three children, and taking care of these and other domestic responsibilities were prioritized over their own health:

One thing is that you are very motivated when pregnant because it depends on you to do the best for your baby. Another thing is when you are completely exhausted as a new mother—you don’t sleep, and there are so many other things to take care of—there is no energy left.

Family responsibilities, resulting in time constraints and lack of resources, were thus barriers to the women's likelihood to break their habits and to start exercising and preparing healthy food. Exercise seemed especially difficult to implement. Some of the women disliked exercise, but most women found it difficult because it challenged their role as primary caregiver. Preparing food was not experienced as an additional time consumer since it was already a part of their domestic responsibilities whereas exercising was viewed as taking time out for themselves away from the family. In addition, attending work meant taking time away from the family making it even more difficult to exercise away from home. Several women called for ideas to incorporate exercise into their daily routines to make it more manageable.

Also over a longer period of time lack of time and energy were barriers to lifestyle changes, as one of the women attending one of the focus groups five years after GDM:

There are new phases all the time. You might start working again, one of the kids start school and another is at the day‐care centre. It’s the new stressful situations you must adapt to and then it's easier to stick to known habits.

Thus, first taking care of their new‐born baby, then other family and domestic responsibilities, and after maternity leave, return to work left minor resources available to change their habits.

#### 3.2.2 Lack of family involvement

The women experienced that GDM and prevention of diabetes in the public and the healthcare system were handled as ‘a women's issue’ rather than a family concern which was a serious barrier to their management of risk factors. Some of the women explained:

Carrots or candy on weekdays? Parents should compromise, but we have rather different stances. I have run out of steam and as a mother I cannot be bothered to be ‘the bogeyman’ all the time.

The diet is kind of difficult. I need to make it fit with the rest of the family—or I have to make my own food. And it’s just not possible for me. Also, if I jog, it would really be motivating if it's not only my responsibility to get out.

The women's discussions reflected that their family's needs took precedence over their individual needs. To meet their caregiver‐roles was an important factor in the women's likelihood to succeed with their lifestyle changes. Lack of support from a partner was considered a barrier to lifestyle modifications, both regarding assistance with household work as indicated in the quotes above, and emotional, as one woman said:

So it’s a bit difficult to find support at home because he [the husband] hasn’t really been involved so far. It [GDM/diabetes] is something I have been responsible for. I’m sure it would facilitate understanding if he knew what it was all about.

#### 3.2.3 Barriers to healthy eating

The women experienced that the social context in which food is embedded was a central barrier to accomplish healthier eating. Food was described an essential part in social gatherings and a vital symbol of hospitality. In combination with the experience of diabetes as a taboo, the women found it difficult to decline food served at social events:

You always eat when you are going out. You want to make an effort for your guest and make them feel welcome—and it is often rich food. You cannot really take the liberty to decline. It is easier at home to say ‘stop’, where no one will notice.

The women experienced a lack of understanding and social pressure from their peers to eat unhealthy food. This was especially significant among the groups of women who were not diagnosed with diabetes. Some women occasionally stayed away from social gatherings to avoid the temptation to eat unhealthy food, but most said that they did not want to isolate themselves. Furthermore, several women described their perception of sweets, cakes and other unhealthy foods as a pleasure and a reward, which was difficult to resist.

#### 3.2.4 Lack of knowledge

Lack of knowledge of consequences of GDM for themselves and their families and of how to incorporate lifestyle adjustments into everyday life constituted barriers towards initiating lifestyle modifications.

The women also indicated insufficient knowledge transfer from healthcare professionals. In Denmark, the responsibility for GDM follow‐up lies with the general practitioner. However, most of the women were not invited for diabetes screening during the postpartum period and they met scanty attention from the healthcare system in contrast to the extensive care they received during their pregnancy:

After the childbirth, the only follow‐up is an appointment with your general practitioner. After being checked all the time during pregnancy, suddenly you feel very alone. It’s like they think that now it’s not serious anymore.

As the quote illustrates, the women were not satisfied with the postpartum support and felt they were on their own with worries regarding prevention of the long‐term consequences of GDM. The deficient knowledge thus leads to feeling insecure regarding how to handle the situation after GDM.

### 3.3 Experiences of motivational factors for prevention strategies

The well‐being of the family in the present and the future appeared as a major motivational factor for lifestyle changes. Some of the women said for example:

After childbirth, I lost a lot of weight because my children should have a mother who is fit to fight. They should have a mother who can play with them and move around.

I think I owe my family and especially my son to try to prevent diabetes. It’s important for me to be a good example. My major motivation is these children.

As the quotes illustrate the women found it important to be a good parent and a role model for their children to prioritize the well‐being of their family. This could mean providing healthy food and taking care of their own health to show the children ‘*how to live a fine and healthy life*’ as some of the women expressed it. They emphasized that knowledge about risk factors for their children and partners would be a highly motivating factor for lifestyle changes:

It is very motivating to be reminded that GDM has an impact also for my daughter and my husband. It is not only for me.

Well, I haven’t learned anything about the risk for my son. I’ve only heard that I risk getting diabetes. Vegetables and fruit instead of candy, I think you would fancy that more if you know it is the whole family who must take care.

The women thus stressed that if the family as a whole were aware of risk factors it would be easier to decide where to put their effort as a family and support each other in lifestyle changes.

Furthermore, a motivating factor to overcome challenges regarding time constraints was to integrate lifestyle changes into the women's everyday life. They explained it would be easier to hold on to the new habits if it fitted into their family obligations, work and social life. They mentioned for example physical activities with their children, but they also specified a need for inspiration to develop such initiatives further. Lastly, social support from friends and extended family appeared as an essential motivating factor.

### 3.4 Suggestions for motivational preventive programs

Based on their experiences with GDM/diabetes and preventive strategies, the women provided suggestions for organizing motivating preventive programs. The most important issues are presented in this section, while Table 4 provides an overview of frameworks for preventive initiatives.

**TABLE 4:** Organization of preventive programs targeting consequences of GDM

Program components	Program framework and substance
Conditions	Basic motivational incentives in the short and long term is the health and well‐being of the child/children and husband/partner The overall quality of life in the family should be the focal point rather than diet and exercise restrictions Family anchored initiatives Participating in the initiative should be free of cost or cheap
Form	The course program is organized as group sessions (ie social and peer support is pivotal for the success of the program) Face‐to‐face meetings approximately monthly for 6–8 sessions Duration over approximately a year Recruitment during pregnancy or 3–6 months after delivery (ie ‘windows of opportunity’) Lessons and support from experienced healthcare professionals The program should be led by a recurring person and involve relevant visiting instructors Follow‐up appointment half a year after ending the course (focus on adherence to lifestyle changes) Opportunity for the participants to meet ‘on their own’ after the course program
Substance	Focus on everyday life, including work life, as foundation for lifestyle changes Focus on lifestyle changes as a joint family affair Focus not only on diet and exercise but also on the overall quality of life and well‐being of the family Greater priority to integrate exercise in everyday life and exercise as a joint family affair Information on breastfeeding's positive effect considering the prevention of diabetes Group‐based activities, for example walking, cooking and eating together Instructions for easy, fast and healthy cooking Individually tailored advice on diets Encompassment of a psychologist/coach focusing on psychological and emotional aspect considering adherence to lifestyle changes
Location	Some transport is not a barrier to participate in a course if the course gives meaning, makes sense and is of good quality If possible, it is preferable to gather women and their families from within a local area because it will be easier for the women to keep in touch with the community when the program ends It is preferable to organize the program under the auspices of local authority (eg the municipality)
Contacts	Invitations to screening for diabetes after GDM Reminders about recurrent check‐ups at general practitioners or hospital/specialized care Preferable personal contact as much as possible, for example when invited to the program

The women strongly suggested family‐based programs rather than targeting solely women. Involving the families were perceived as time‐saving and targeting risk factors in the family as a whole would make it easier to adhere to lifestyle changes. Further, the women suggested group classes by highlighting the importance of sharing experiences with people with similar challenges. They advocated for face‐to‐face meetings compared to virtual meetings because socializing was perceived as pivotal for sharing experiences and support. The women suggested to increase the focus on exercise. They recommended tailoring diet counselling to individual families’ preferences. Overall, the women strongly advocated for an approach based on the family's quality of everyday life instead of directions, rules and ‘*being blamed*’, as they described it.

Timing was an important factor. Three to six months after birth, alternatively during pregnancy, seemed to be ‘a window of opportunity’, a period when the stress in their everyday life with a new‐born child was somehow settled, and they were still on maternity leave (according to Danish regulations) with more resources regarding time, energy and motivation to initiate new lifestyle habits. The women interviewed five years after their GDM diagnosis expressed similar attitudes to timeframes, but they furthermore emphasized that participating in preventive programs continued to be important because they still felt left alone with their concerns about the possible consequences of GDM. In addition, the women diagnosed with diabetes indicated a strong need for more support.

The women emphasized the importance of long‐lasting initiatives and peer support to succeed with new lifestyle habits. They suggested meeting 6–8 times over a year and possibilities to meet within the group after the formal end of the course, without the presence of a healthcare professional.

Finally, the women highlighted the importance of the program being managed by experienced healthcare professionals. The women preferred longer transport as long as the programme was led by competent healthcare professionals.

## 4 CONCLUSIONS

The study revealed three main findings. Firstly, being diagnosed with GDM was an upsetting experience and the women were especially worried about their unborn baby. Over the five‐year period, the study covered the women experienced continued worries about the possible consequences of GDM. Secondly, the women were highly motivated to prevent consequences of GDM, including the group of women five years after GDM, but they faced major challenges to adhere to the recommended lifestyle changes. The barriers experienced as demotivation factors were lack of time and resources, too little family involvement, lack of knowledge and barriers to healthy eating habits. Thirdly, a powerful motivational factor for complying with the preventive strategies was the well‐being of their children and partners. Also, form and substance of preventive programmes should fit with their everyday life and the family's quality of life. Thus, the family was an important context to the women's attitudes to and possibilities for incorporating preventive steps in their everyday life.

Strength of the study is the long‐term perspective of women's experiences, showing that their worries about the consequences of GDM and awareness of preventive initiatives continued beyond GDM. Moreover, the design using focus group interviews with the participating women representing a range of number of pregnancies, experiences with GDM, broad duration from the onset of GDM and diabetes status, allowed accumulation of broad and detailed knowledge based on the target group's perspectives. This follows an important purpose of the study to initiate a preventive program targeting women's needs in various stages post‐GDM. Therefore, all themes across the groups of women were identified, including opposing views. The study fulfilled most of the items of the COREQ checklist^23^ apart from items regarding the interview transcripts and findings, which were not discussed with the participants. Also the item regarding datasaturation was not discussed since the number of focus group interview were decided upon designing the study. However, themes were very similar across the focus groups, thus indicating datasaturation. A weakness of the study is the relatively large drop‐out rate of the invited participants. Some indicated lack of resources or poor knowledge of Danish when they refused the invitation. These women potentially represent disadvantaged people whose perspectives are relevant for appropriate organization of preventive programs. Also very few women below 30 years participated. They might have different experiences and needs regarding preventive initiatives. There is therefore a need for future research to include such groups. Furthermore, a lack of socio‐demographic information characterising the participating women is a limitation in relation to discussions of transferability of the results. However, the concordance of the main results with previous studies suggests that the results are generally usable. For example, other studies also report intense reactions among the women diagnosed with GDM with their major concerns being the foetus and emotional stress, blaming themselves and feeling stigmatized.^12^, ^13^, ^14^, ^24^, ^25^ Likewise, studies reported women's lack of knowledge about the long‐term risks, also for their offspring and partners, as well as a lack of support.^25^, ^26^, ^27^ Our study thus both strengthened and supplemented existing evidence by showing how these issues were emphasized by women up to five years after their GDM diagnosis, including women with later onset of diabetes. This finding points to the long‐term influence of the diagnosis and highlights the importance of paying attention to women with a history of GDM for a long time.

The women were motivated to preventive initiatives, but experienced barriers for complying, which is found in other studies as well.^12^, ^20^, ^24^, ^25^, ^27^ Also, the family was shown to constitute a pivotal context for preventive programs^13^, ^18^ as well as the importance of the early onset of preventive support from qualified healthcare professionals.^2^, ^15^, ^28^ Interestingly, evidence for eHealth in lifestyle modification is reported by other studies (van den^29^, ^30^ while this study found a strong preference for face‐to‐face interactions with healthcare professionals as well as peers and eHealth as a supplement. This result was also supported by another study.^12^ While other studies found the women being motivated during pregnancy, this study supplements by showing that women were highly motivated to participate in preventive programs over time. Across the 5‐year timespan, the women expressed the same worries concerning consequences,difficulties in complying with preventive strategies,and motivational factors and suggestions to the organization of preventive programs.

Thus, this study provides two important recommendations: the need to be responsiveness to the women and their families even a long time after the onset of GDM; and to frame preventive programs within a holistic approach for the affected families with a main focus on the quality of their everyday life compared to the women's experiences of being stigmatized due to their ‘wrong’ lifestyle habits. Coping strategies should therefore be rooted in the women's perception of GDM/diabetes and based on their experiences with barriers and motivational factors. The well‐being and the quality of life within the family are dominant motivational factors which offer powerful potentials for supporting their coping capability.

## AUTHORS CONTRIBUTIONS

All authors have given final approval of the version to be published. Lisbeth Ørtenblad: contributed substantially to conception and design, acquisition of data collection, conducting analysis and interpretation of data; writing the manuscript and revising it critically. She is the guarantor for the work and the conduction of the study, incl. the decision to publish the work. Diana Høtoft: contributed substantially to acquisition of data collection, interpretation of data; critically revising the manuscript for important intellectual content. Rubab H Krogh: involved in substantial contribution to conception; critically revising the manuscript for important intellectual content. Vibeke Lynggaard: involved in substantial contribution to critically interpretation of the data; revised the manuscript for important intellectual content. Jens Juel Christiansen: involved in substantial contribution to conception; revision of the manuscript. Claus Vinther Nielsen: involved in substantial contribution to conception and design, interpretation of data; revising the manuscript critically for important intellectual content. Anne‐Mette Hedeager Momsen: involved in substantial contribution to conception and design, processing and interpretation of data; drafting the article and revising it critically for important intellectual content.

## Data Availability

No further data than presented in this manuscript is available.
